# Reconstruction and Differential Expression Profiling Core Target Analyses of the circRNA-miRNA-mRNA Network Based on Competitive Endogenous RNAs in Ulcerative Colitis

**DOI:** 10.1155/2022/4572181

**Published:** 2022-10-21

**Authors:** Sai Xu, Shouqiang Chen, Menghe Zhang, Wenrong An, Jie Li, Zhenhai Sun, Yunsheng Xu

**Affiliations:** ^1^Shandong University of Traditional Chinese Medicine, Jinan, China; ^2^Second Affiliated Hospital of Shandong University of TCM, Jinan, China

## Abstract

Ulcerative colitis (UC) is a common autoimmune disease worldwide. Circular RNA (circRNA) is a type of noncoding ribonucleic acids (ncRNAs). In addition to their roles in numerous biological processes, circRNAs are also linked to a vast range of diseases including UC. Although previous studies have examined many circRNAs, the physiological and pathological roles of the circRNA-associated competing endogenous RNA (ceRNA) network in UC remain unclear. Thus, we constructed a circRNA-miRNA-mRNA network based on the ceRNA hypothesis by analyzing data from the National Center for Biotechnology Information Gene Expression Omnibus (NCBI-GEO) database. Genes with higher degree values than others in the ceRNA network were selected as central nodes when constructing the corresponding core subnetworks. To fully understand the biological function of the ceRNA network, we entered all differentially expressed mRNAs (DEmRNAs) from the ceRNA network into the Database for Annotation and Integrated Discovery (DAVID), which was used to perform Gene Ontology (GO) and Kyoto Encyclopedia of Genes and Genomes (KEGG) enrichment analyses. We further entered DEmRNAs into the STRING database for protein-protein interaction (PPI) network analysis. The results elucidated that the ceRNA network comprised 403 circRNA nodes, 5 miRNA nodes, 138 mRNA nodes, and 559 edges. Three core ceRNA subnetworks centered on hsa-miR-342-3p, hsa-miR-199a-5p, and hsa-miR-142-3p were reconstructed in this study. GO and KEGG enrichment analyses identified 167 enriched GO categories and 14 enriched KEGG pathway terms. The core PPI network was composed of 15 core targets, of which CD44, HIF1A, and MMP2 were the most significant. In summary, 3 hub miRNAs (hsa-miR-342-3p, hsa-miR-199a-5p, hsa-miR-142-3p) and 3 hub genes (CD44, HIF1A, and MMP2) might play an important role in the development of UC. These hub nodes, first proposed here, might also be used as potential diagnostic markers and therapeutic targets.

## 1. Introduction

Ulcerative colitis (UC) is one of the major types of inflammatory bowel disease (IBD) and is a common autoimmune disease worldwide. Colorectal cancer (CRC) develops if the disease deteriorates [1–3]. The notably consistent features of UC are characterized by chronic uninterrupted inflammation of the intestinal mucosa, persistent infections in the affected tissue, bleeding, diarrhea, bowel urgency, and abdominal pain [4]. Often, the disease course follows an alternating cycle of exacerbation and remission [5]. The prevalence of UC varies considerably across countries and is higher in Western countries, particularly in Europe and North America [6]. According to epidemiological studies, the highest incidence of UC is 57.9/100,000 persons/year in Northern Europe and 23.14/100,000 persons/year in North America [7]. Since the end of the last century, a steep increase in the occurrence of UC in low-incidence areas such as Asia has been observed [8]. Indeed, a number of diseases are associated with UC, such as rheumatoid arthritis and interstitial nephritis [9, 10]. This disease is endemic and prevalent worldwide, imposing substantial health and economic burdens in many countries due to lost labor and high costs to healthcare systems [11–13].

The mechanisms controlling the pathophysiological processes of UC are extremely complex and the exact mechanism of its pathogenesis is still unclear [14, 15]. Recently, it has been suggested that UC is associated with the dysregulation of the host's mucosal immune system, environmental factors, changes in the intestinal microbiome, and genetic susceptibilities [16–18]. To identify therapeutic strategies for reducing the incidence of UC, it is imperative to investigate UC-specific molecular pathogenesis and look for a novel and curative treatment.

Noncoding ribonucleic acids (ncRNAs) are RNA molecules that lack the ability to encode proteins. NcRNAs exist in several subtypes, microRNAs (miRNAs), circular RNAs (circRNAs), small nucleolar RNAs (snoRNAs), small nuclear RNAs (snRNAs), and transfer RNAs (tRNAs) are among them. Identified as a class of small noncoding RNAs, miRNAs are short (18–25 nucleotides in length), endogenous, noncoding, and single-stranded RNAs that are highly conserved. Even though they do not have the ability to encode proteins, they can negatively regulate gene expression [19]. miRNAs affect several pathophysiological processes, such as cell division, apoptosis, immune response, and tissue homeostasis [20]. circRNAs are covalently closed RNA transcripts (single-stranded) that participate in human diseases by regulating gene expression [21]. A circRNA has a specific circular structure and is produced by a “back-splicing” mechanism [22]. In humans, circRNAs play a significant role in regulating gene expression to participate in diseases, but their functions in UC remain controversial [21].

According to the competing endogenous RNA (ceRNA) hypothesis, circRNAs, miRNAs, and messenger RNAs (mRNAs) form an intricate network of regulatory molecules. circRNAs may play pivotal roles in gene regulation. For example, circRNAs act as miRNA “sponges” to release miRNAs and target mRNAs [23, 24]. Nonetheless, there have been few studies on the interactions of the circRNA-miRNA-mRNA network in UC. In light of the fact that genetic factors play a crucial role in the pathogenesis of UC, it will be beneficial to strengthen our understanding of the relationships between circRNAs, miRNAs, and target genes to learn more about UC mechanisms and identify novel biomarkers.

**Figure 1:**
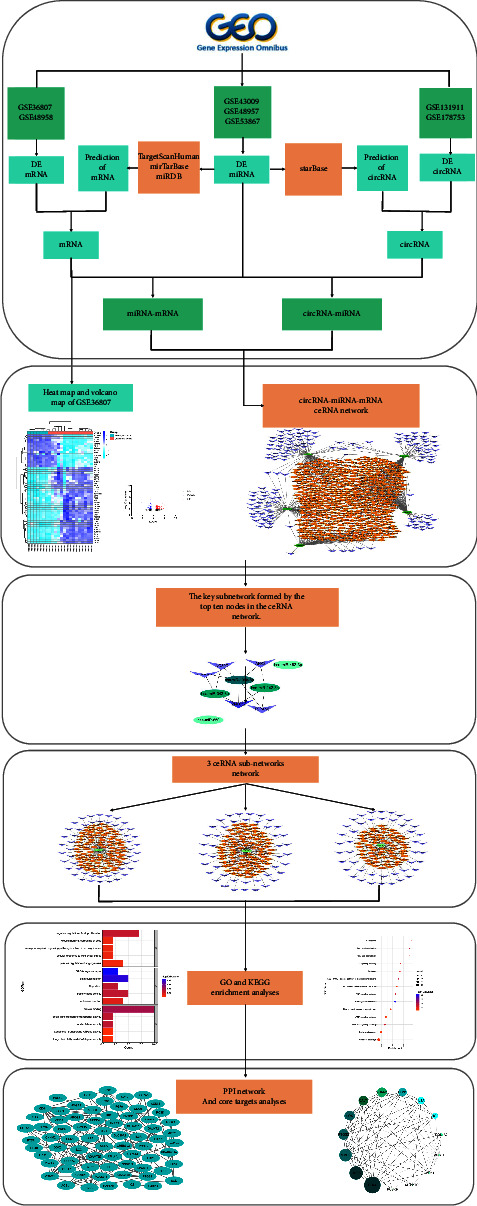
The workflow for the circRNA-associated ceRNA network analysis in ulcerative colitis.

In the present study, an extensive analysis of circRNA, miRNA, and mRNA expression profiles in UC patients and healthy individuals was performed. Based on the ceRNA hypothesis, we constructed a circRNA-miRNA-mRNA triple network and the core ceRNA subnetworks were reconstructed. After that, we examined pathways linked to significant differentially expressed genes (DEGs) based on Gene Ontology (GO) and Kyoto Encyclopedia of Genes and Genomes (KEGG) analyses. Finally, protein-protein interaction (PPI) network analysis was conducted on differentially expressed mRNAs (DEmRNAs). Through this study, we enhanced our understanding of the role of the circRNA-associated ceRNA network in UC pathogenesis and obtained valuable insights for further research. The workflow for the circRNA-associated ceRNA network analysis in UC is shown in Figure 1.

## 2. Materials and Methods

### 2.1. Raw Data

MRNAs (GSE36807, GSE48958), miRNAs (GSE43009, GSE48957, and GSE53867), and circRNAs (GSE131911, GSE178753) corresponding to the clinical information of UC patients were freely accessible from the National Center for Biotechnology Information Gene Expression Omnibus (NCBI-GEO) (https://www.ncbi.nlm.nih.gov/geo/#). GEO is a public functional genomic data repository that accepts microarray and sequencing data. It also provides tools to help users query and download experimental gene expression profiles. The data set utilized in our study included 109 samples, of which 43 were healthy human control samples and 66 were UC colonic samples. Microarray data can be used for mass screening to detect key differentially expressed genes (circRNAs, mRNAs, and miRNAs). We downloaded UC circRNA, miRNA, and mRNA microarray data from the GEO database for the corresponding research.

### 2.2. Screening of Differentially Expressed circRNAs, miRNAs, and mRNAs

After background correction and matrix data normalization for microarray data downloaded from the GEO database, the differentially expressed circRNAs (DEcircRNAs), differentially expressed miRNAs (DEmiRNAs), and DEmRNAs between UC patients and healthy controls were calculated. The DEGs (DEcircRNAs, DEmiRNAs, DEmRNAs) from all data sets with |logFC, log2 fold change| ≥ 1.0 and *p* < 0.05 after correction were considered to satisfy the selection criteria for subsequent analysis. Thereafter, the intersections of the DEmRNAs, DEmiRNAs, and DEcircRNAs on the microarray were obtained, respectively. A logFC of ≥1.0 indicated that DEGs were upregulated in UC; a logFC of ≤ −1.0 indicated downregulation in UC. Both a heatmap and a volcano plot of the data in GSE36807 were constructed for visualization.

### 2.3. Prediction of Target circRNAs and mRNAs of Differentially Expressed miRNAs

First, it should be stated that combining with the DEmiRNAs, we predicted the integrated circRNA-miRNA pairs using starBase (https://starbase.sysu.edu.cn/). Second, predictions of target mRNAs related to DEmiRNAs were retrieved from the following three databases: TargetScanHuman (https://www.targetscan.org/vert_72/), miRTarBase (https://mirtarbase.cuhk.edu.cn/), and miRDB (https://mirdb.org/). Finally, we established matched circRNA-miRNA pairs and miRNA-mRNA pairs.

### 2.4. Construction of the circRNA-miRNA-mRNA Competing Endogenous RNA Network

The circRNA-miRNA-mRNA ceRNA network was established by recombining all co-expressed circRNA-miRNA pairs as well as miRNA-mRNA pairs and was visualized using Cytoscape 3.7.2 software. Simultaneously, all node degrees in the ceRNA network were calculated and the subnetworks of the circRNA-associated ceRNA network were then estimated.

### 2.5. Reconstruction of the Key circRNA-miRNA-mRNA Subnetworks

We further reconstructed the key subnetworks by comparing the node degrees of genes along with their corresponding circRNA-miRNA and miRNA-mRNA pairs in the holistic ceRNA network. Reconstruction of key subnetworks was performed using the Cytoscape 3.7.2 plug-in CytoHubba [25]. This plug-in is used to identify the key targets and subnetworks of complex networks. In addition, the plug-in can calculate the information of each node in the network diagram. According to recent research studies, the top 25% of nodes in the ceRNA network can be selected [26], and only the complete circRNA-miRNA-mRNA axes were retained.

### 2.6. Functional Enrichment Analyses

To assess functional enrichment, the Database for Annotation and Integrated Discovery (DAVID, https://david.ncifcrf.gov/) was utilized in the analysis of DEmRNAs. We entered all DEmRNAs from the circRNA-associated ceRNA network into the DAVID database, querying and acquiring the biological processes in GO and KEGG (*p* < 0.05). Bioinformatics software (https://www.bioinformatics.com.cn) was ultimately used for visualizing the results of GO and KEGG functional enrichment analyses of the DEmRNAs.

### 2.7. Building of the Protein-Protein Interaction (PPI) Network

The PPI network was constructed using the STRING online database (STRING, https://string-db.org/). We input DEmRNAs within the circRNA-associated ceRNA network into the STRING database and the corresponding PPI network was thus obtained. Afterward, the constructed PPI network was visualized by using the CytoHubba plug-in in Cytoscape 3.7.2. In a previous study, the nodes with degree values > 5 in the network were defined as the hub genes in the PPI regulatory network [27]. Thus, we rebuilt a core PPI network based on these degree values.

## 3. Results

### 3.1. Differentially Expressed circRNAs, miRNAs, and mRNAs

**Figure 2:**
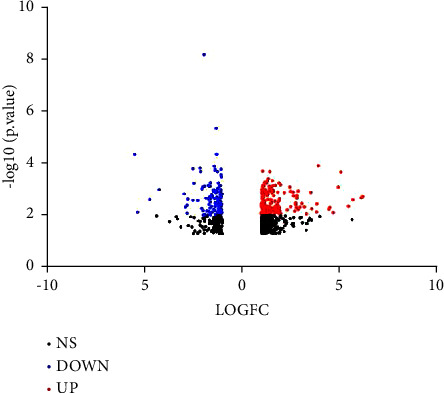
Volcano plot of DEmRNAs from GSE36807 of UC. Red represents upregulated DEmRNAs and blue represents downregulated DEmRNAs.

**Figure 3:**
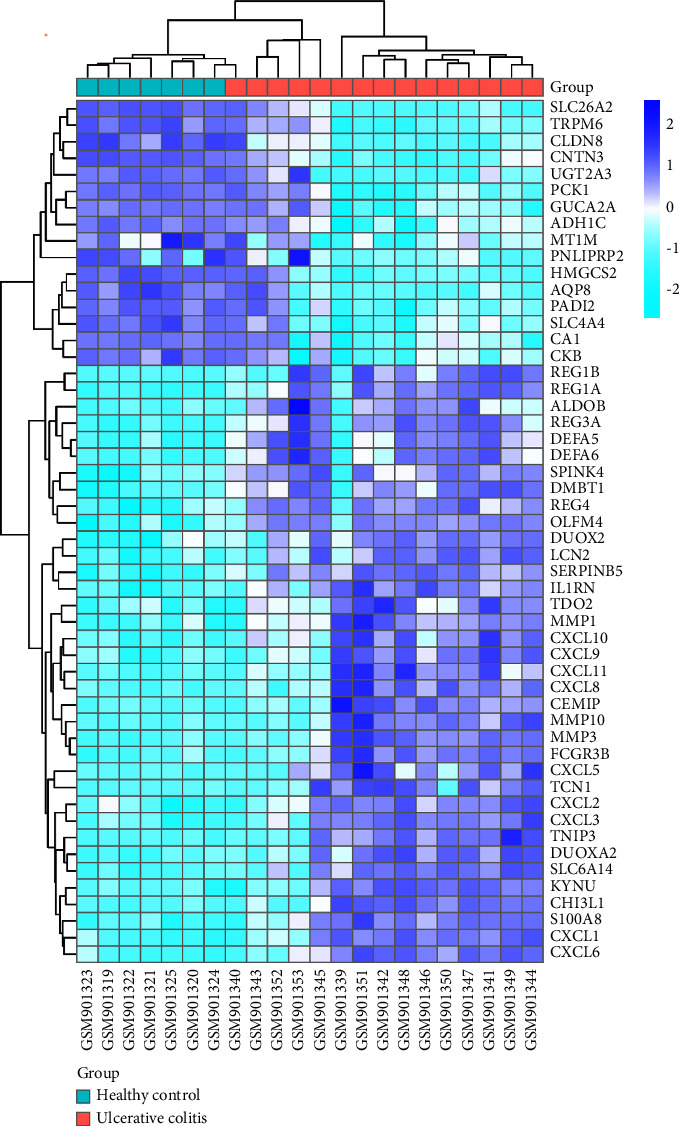
Heatmap of DEmRNAs from GSE36807 of UC. Purple boxes represent upregulated DEmRNAs and blue boxes represent downregulated DEmRNAs. Pink and blue, respectively, represent UC and healthy control.

Seven microarray datasets (GSE36807, GSE48958, GSE43009, GSE48957, GSE53867, GSE131911, and GSE178753) were analyzed in this study. The expression profiles of mRNAs, miRNAs, and circRNAs in 66 UC samples and 43 normal samples were calculated in total. Preprocessing of the raw data revealed 1809 DEGs which included 878 DEmRNAs, 881 DEcircRNAs, and 50 DEmiRNAs (|logFC, log2 fold change| ≥ 1.0, and *p* < 0.05). Of these DEGs, 499 DEmRNAs, 460 DEcircRNAs, and 14 DEmiRNAs were upregulated and 379 DEmRNAs, 421 DEcircRNAs, and 36 DEmiRNAs were downregulated. We took microarray data GSE36807 as a representative constructed for visualization, including 676 DEmRNAs. The volcano plot (|logFC, log2 fold change| ≥ 1.0, and *p* < 0.01) and heatmap of the DEmRNAs in GSE36807 are shown in Figures 2 and 3.

### 3.2. Establishment of miRNA-mRNA and circRNA-mRNA Interactions

The TargetScan, miRDB, and miRTarBase databases were, respectively, used to predict the target mRNAs of the 50 DEmiRNAs. The results obtained from the three databases were integrated. A total of 5954 miRNA-mRNA pairs were acquired after removing duplicates. Then, the mRNAs in these 5954 pairs were intersected with the 878 DEmRNAs, successfully attaining 285 pairs of miRNA-mRNA interactions consisting of 13 DEmiRNAs and 209 DEmRNAs. Through the same procedure, the starBase database was used to successfully measure the circRNAs targeting the 50 DEmiRNAs, harvesting 403 pairs of circRNA-mRNA interactions comprising 5 DEmiRNAs and 403 DEcircRNAs. The results received from the database intersected with the 881 DEcircRNAs. Since no intersecting circRNA-mRNA pairs were found, the ceRNA network was constructed using the 403 circRNA-mRNA pairs predicted by starBase.

### 3.3. Construction of the Competing Endogenous RNA Network

**Figure 4:**
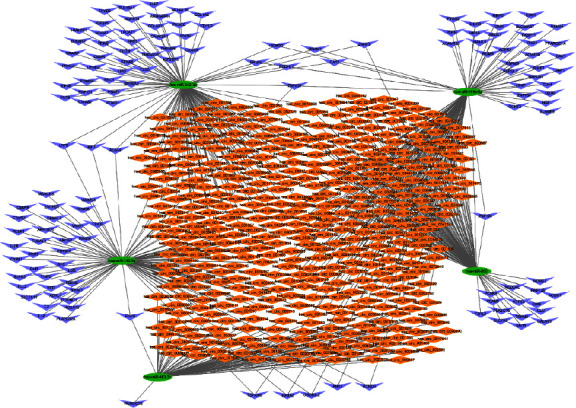
The UC-related circRNA-mRNA-miRNA ceRNA regulatory network. Orange diamond represents circRNAs, green oval represents miRNAs, and purple arrow represents mRNAs.

To better understand the roles of DEcircRNAs in UC and the interactions between these DEcircRNAs and DEmiRNAs, we established a circRNA-miRNA-mRNA ceRNA network of UC. All circRNA-miRNA and miRNA-mRNA pairs were integrated, and Cytoscape 3.7.2 was further adopted for constructing and visualizing the circRNA-miRNA-mRNA ceRNA network. As shown in Figure 4, the circRNA-associated ceRNA network was composed of 403 circRNA nodes, 5 miRNA nodes, 138 mRNA nodes, and 559 edges.

### 3.4. Reconstruction of the Key circRNA-miRNA-mRNA Subnetworks

**Figure 5:**
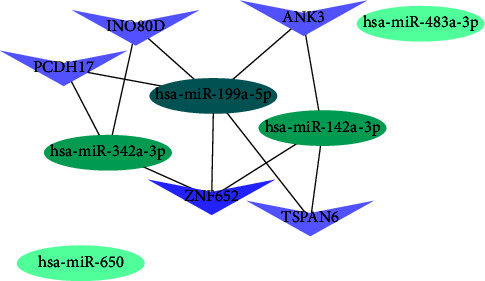
The top ten nodes in the circRNA-associated ceRNA network. Oval represents miRNAs, arrow represents mRNAs, nodes with darker colors are more important.

**Figure 6:**
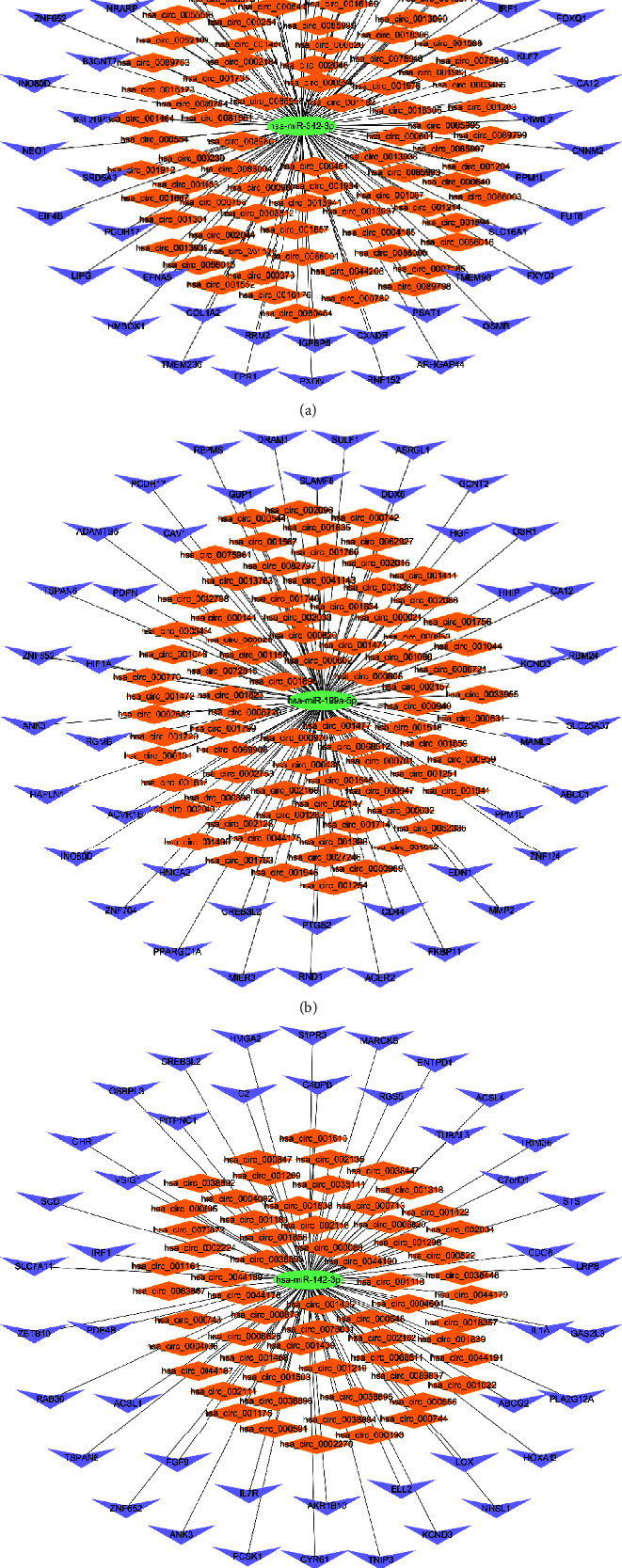
Three key circRNA-associated subnetworks. (a) The key subnetwork of circRNA-hsa-miR-342-3p-mRNA. (b) The key subnetwork of circRNA-hsa-miR-199a-5p-mRNA. (c) The key subnetwork of circRNA-hsa-miR-142-3p-mRNA.

For further identification of hub genes as well as their related networks, the Cytoscape 3.7.2 plug-in CytoHubba was utilized for calculation and visualization of all node degree values in the circRNA-associated ceRNA network. We selected the top 10 nodes ranked by degree value, and because the number of nodes selected was far less than the top 25% of initial nodes in the ceRNA network, the 10 selected nodes represented more accuracy. The top 10 nodes were hsa-miR-342-3p, hsa-miR-199a-5p, hsa-miR-650, hsa-miR-142-3p, hsa-miR-483-3p, ZNF652, INO80D, ANK3, TSPAN6, and PCDH17. The key network formed by the top 10 nodes is shown in Figure 5. Specifically, hsa-miR-342-3p, hsa-miR-199a-5p, hsa-miR-650, hsa-miR-142-3p, and hsa-miR-483-3p were five of the top-ranked nodes. It was regretful that hsa-miR-650 and hsa-miR-483-3p were not interconnected with the key network, so we eliminated these two miRNAs. Ultimately, 3 new key subnetworks were reconstructed. Three miRNAs that served as the center of the subnetwork significantly regulated transcription. The 3 key subnetworks of circRNA-hsa-miR-342-3p-mRNA, circRNA-hsa-miR-199a-5p-mRNA, and circRNA-hsa-miR-142-3p-mRNA were extracted, as shown in Figures 6(a)–6(c). The key subnetwork of circRNA-hsa-miR-342-3p-mRNA comprised 1 miRNA node, 50 mRNA nodes as well as 89 circRNA nodes (Figure 6(a)), the key subnetwork of circRNA-hsa-miR-199a-5p-mRNA consisted of 1 miRNA node, 43 mRNA nodes and 81 circRNA nodes (Figure 6(b)), the key subnetwork of circRNA-hsa-miR-142-3p-mRNA was composed of 1 miRNA node, 44 mRNA nodes along with 62 circRNA nodes (Figure 6(c)).

### 3.5. Functional Enrichment Analyses of Differentially Expressed mRNAs

**Figure 7:**
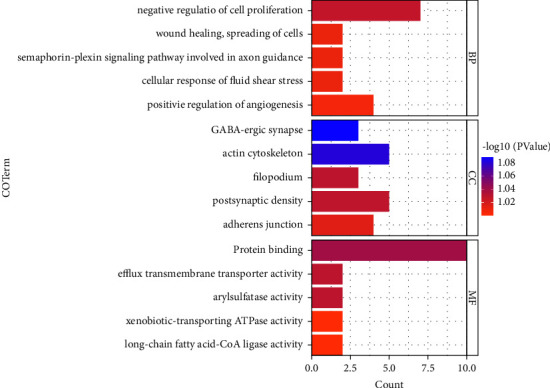
The top 5 GO terms in each section (BP, CC, and MF) are displayed as a bar diagram by analyzing DEmRNAs. The colors indicate different −log_10_ (*p* value).

**Figure 8:**
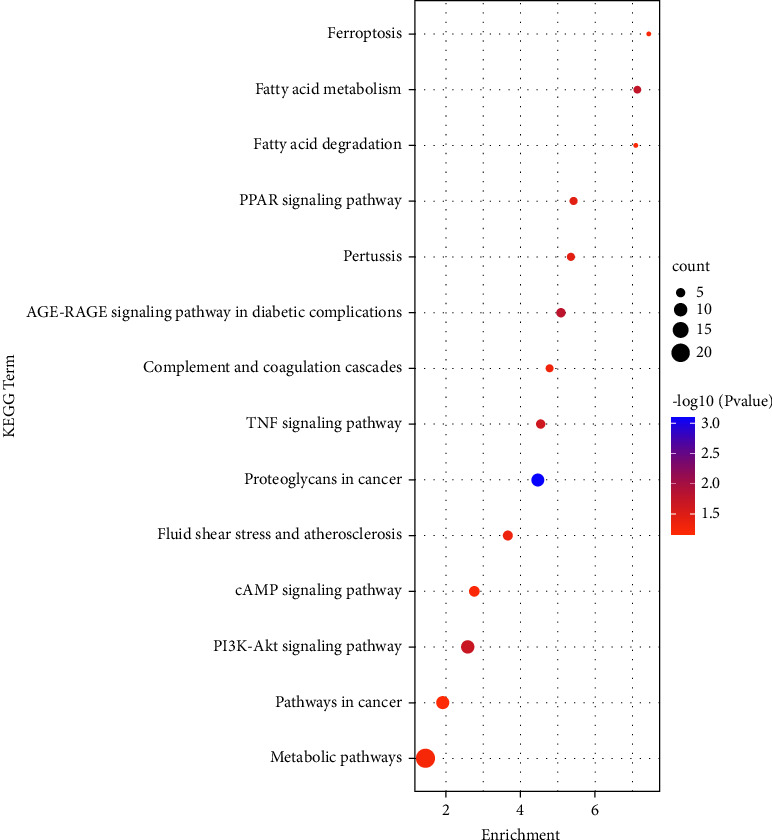
The total 14 KEGG pathway terms are displayed as a bubble diagram by analyzing DEmRNAs. The colors indicate different −log_10_ (*p* value) and the sizes of the circle represent counts.

Analyses of the functions of 138 DEmRNAs, which came from the circRNA-associated ceRNA network, were carried out. The results elucidated the enrichment of 167 GO terms that occurred in biological processes, namely, 115 biological process (BP) terms, 36 cellular component (CC) terms, and 16 molecular function (MF) terms, with a threshold value of *p* < 0.05. The top 5 significant GO terms in each section are shown in Table 1 and Figure 7. At the biological process (BP) level, the DEmRNAs participated principally in the following terms: positive regulation of angiogenesis, cellular response to fluid shear stress, semaphorin-plexin signaling pathway involved in axon guidance, wound healing, spreading of cells, and negative regulation of cell proliferation. In general, at the cellular component (CC) level, the DEmRNAs were affiliated with adherens junctions, postsynaptic density, filopodium, actin cytoskeleton, and GABAergic synapses. Most of the DEmRNAs enriched in molecular function (MF) terms were associated with long-chain fatty acid-CoA ligase activity, xenobiotic-transporting ATPase activity, arylsulfatase activity, efflux transmembrane transporter activity, and protein binding. Subsequently, KEGG pathway enrichment analysis of all DEmRNAs in the circRNA-associated ceRNA network was implemented. The 14 identified KEGG pathways are demonstrated in Table 2. As shown in Figure 8, the genes were significantly enriched in 7 pathways, namely, pathways in cancer, cAMP signaling pathway, metabolic pathways, PPAR signaling pathway, TNF signaling pathway, PI3K-Akt signaling pathway, and AGE-RAGE signaling pathway in diabetic complications. As mentioned above, these results proved that DEmRNAs play crucial roles in a number of biological processes and functions, such as the inflammatory response and immune response, which are crucial in the occurrence and development of UC.

### 3.6. Building of the Protein-Protein Interaction (PPI) Network of Differentially Expressed mRNAs

**Figure 9:**
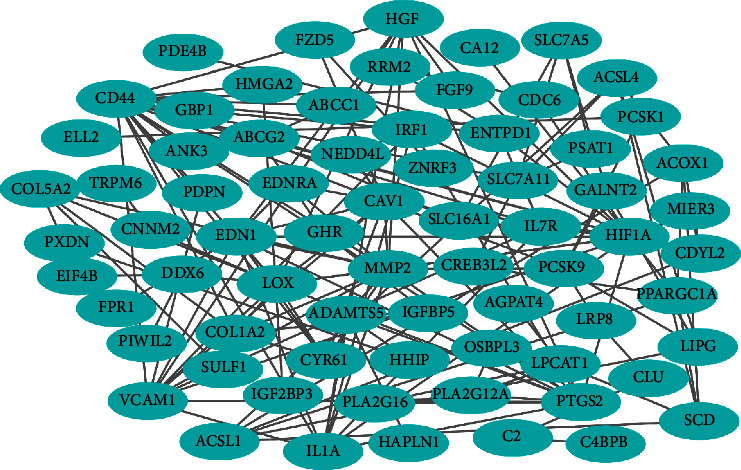
The PPI network of 71 targets of DEmRNAs. Edges represent protein-protein associations.

**Figure 10:**
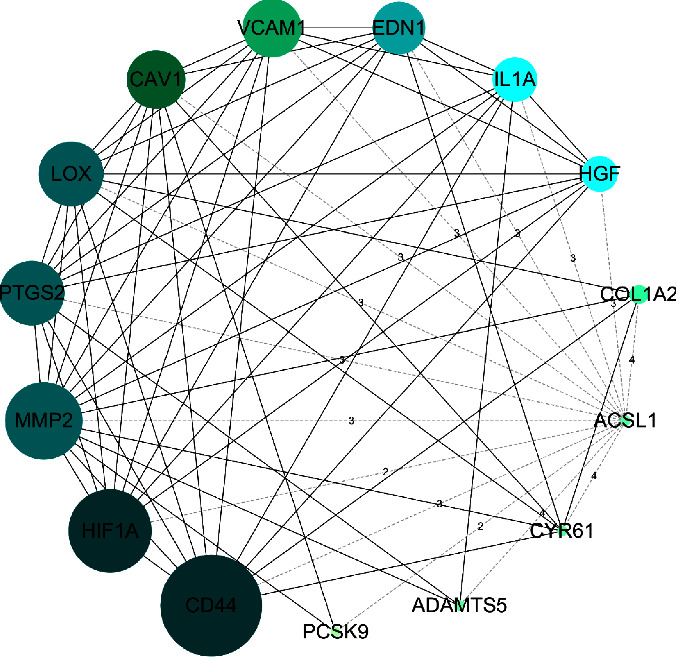
PPI network of 15 core targets. The degree value > 5. The node size from small to large and node color from light green to dark green represent the degree value from low to high.

We input the DEmRNAs in the circRNA-associated ceRNA network into the STRING database to build a PPI network, which contained 71 nodes and 150 edges, as shown in Figure 9. Thereafter, this PPI network was analyzed by the CytoHubba plug-in in Cytoscape 3.7.2. According to the degree value obtained by the topological analysis, the top 15 nodes (degree value > 5) in the PPI network were further selected as the core targets: CD44, HIF1A, MMP2, PTGS2, LOX, CAV1, VCAM1, EDN1, IL1A, HGF, COL1A2, ACSL1, CYR61, ADAMTS5, and PCSK9. We reconstructed a PPI network graphic of the 15 core targets based on the ceRNA network, as shown in Figure 10. The core PPI network consisted of 15 nodes and 69 edges. Nodes are represented by circles; the node size ranges from small to large and the node color from light to dark represents the degree value from low to high. The higher the value, the more important is the target. Among the nodes, CD44 had the highest degree value (degree = 19), followed by HIF1A (degree = 16) and MMP2 (degree = 15). Thus, these 3 targets are the most significant core targets in the ceRNA network associated with UC and should be considered for further research.

## 4. Discussion

In the present study, we integrated microarray data from circRNA, miRNA, and mRNA expression profiles in human colon tissue. With |logFC| ≥ 1.0 and *p* < 0.05 as the thresholds, the expression of 460 upregulated and 421 downregulated circRNAs, 14 upregulated and 36 downregulated miRNAs, along with 499 upregulated and 379 downregulated mRNAs showed significant differences between the UC patients and the control individuals. As the genes enumerated above were abnormally expressed in UC, we speculated that these genes are involved in UC pathogenesis and development. For instance, RNU2-1 is considered a potential diagnostic biomarker for pancreatic and colorectal adenocarcinoma [28]. In this study, RNU2-1 was upregulated in UC, which indicated that UC patients were more prone to colon cancer. Alterations in the DPP10 level can modify the inflammatory responses of lung epithelial cells [29]. However, in this research study, DPP10 was downregulated in UC, suggesting a role for the inflammatory process in this disease.

A new principle underlying RNA interactions has been revealed by the ceRNA hypothesis, which is as follows: circRNAs in complex ceRNA regulatory networks competitively bind to miRNAs to affect gene silencing by miRNAs, to participate in gene regulation, and to regulate mRNA expression. Molecules in the ceRNA network are in a state of equilibrium during normal physiology, once that balance is disturbed, disease occurs [30]. As a consequence, the role of circRNA-associated ceRNA networks and their regulatory mechanisms are of immense significance. Although ceRNA networks corresponding to atrial fibrillation, heart failure, and colon cancer have been constructed in numerous studies [31–33], little information is available on the UC-related ceRNA network. In this research, we innovatively generated a characteristic circRNA-miRNA-mRNA network associated with UC, which was composed of 403 circRNA nodes, 5 miRNA nodes, 138 mRNA nodes, and 559 edges. We also identified nodes called hub nodes, which have been previously shown to be characterized by their high degree of interconnection with other nodes and can be used as topological properties of the network to determine the important genes [34, 35]. As a result of extracting the subnetworks in the circRNA-associated ceRNA network, we identified the following 3 key genes: hsa-miR-342-3p, hsa-miR-199a-5p, and hsa-miR-142-3p. circRNA-miRNA pairs associated with the key genes can be used as diagnostic biomarkers of UC and have irreplaceable implications.

As the central elements of the ceRNA network, miRNAs appear to be crucial to RNA transcription crosstalk. miR-342-3p, miR-199a-5p, and miR-142-3p are three substantial miRNAs implicated in diverse disease pathways. miR-342-3p plays a role in the development of inflammation, which is associated with chronic diseases. A report has indicated that the overexpression of miR-342-3p following inhibition of NEAT1 decreases the release of the inflammatory cytokines IL-6, IL-1*β*, TNF-*α*, and cyclooxygenase-2 in type 1 diabetes mellitus [36]. miR-342-3p expression has been observed to be decreased in the rectosigmoid area in UC patients and is implicated in mediating inflammation and cancer processes [37]. miR-342 has also been demonstrated to act as a tumor suppressor gene that inhibits the growth of colorectal carcinoma by regulating aberrant DNA hypermethylation [38]. Currently, miR-199a-5p is primarily being investigated in cancers, including epithelial ovarian cancer and lung cancer [39, 40]. Pathways linked to miR-199a-5p have also been implicated in the inflammatory response to certain diseases. miR-199a-5p levels are elevated in both patients with Crohn's disease (CD) and patients with UC compared to healthy controls [41]. These observations support the idea that miR-199-5a is involved in the inflammatory process that is common in IBD and other diseases. miR-142-3p is a novel inflammatory regulator that controls proinflammatory mediators, inhibits apoptosis, and may be associated with inflammatory processes in multiple diseases [42]. According to a previous study, miR-142-3p levels in mixed saliva samples are significantly higher in UC cases than in control cases, but there are no noticeable changes in CD cases [43]. miR-142-3p is therefore involved in the pathological process of UC and is also a good diagnostic indicator of UC and CD. Additionally, miR-142-3p acts as an epigenetic regulator and plays a different role in tumorigenesis. miR-142-39 is not just a tumor suppressor in gastric cancer [44], it is also a tumor promoter that exacerbates the development of colorectal cancer [45].

miR-342-3p, miR-199a-5p, and miR-142-3p are interconnected and unique. While all three affect different diseases and cancer, they are also implicated in the inflammation and pathogeneses of UC. As of right now, each of these three miRNAs has been the subject of several studies, but the circRNA-associated ceRNA network shaped by each miRNA has received little attention. In the present study, we established a circRNA-associated ceRNA network of UC and 3 core subnetworks: the circRNA-hsa-miR-342-3p-mRNA network, circRNhsa-miR-199a-5p-mRNA network, and circRNA-hsa-miR-142-3p-mRNA network. This study is the first to show that miR-342-3p, miR-199a-5p, and miR-142-3p coexist in a circRNA-associated ceRNA network and that each miRNA individually comprises a corresponding core subnetwork. Since all three miRNAs participate in the regulation of UC inflammation, it is speculated that the entire ceRNA network and the three core subnetworks are directly correlated with the occurrence and development of UC, particularly with inflammation and carcinogenesis. Owing to differences in specific miRNA expression being among the factors that can increase the risk of colorectal cancer [37], we should plan to further understand the pathological mechanism of UC by focusing on genes in the core ceRNA networks and striving to find a regulatory pathway that is inextricably linked to UC. Then, interventional treatment can be administered to prevent cancerization and further spread of UC.

Analyses of GO terms and KEGG pathways allowed us to better understand the biological functions of downstream mRNAs. As a result of GO enrichment analysis, we identified numerous genes involved in cell proliferation and apoptosis, immune responses, intercellular communication, and signal transmission. Furthermore, KEGG pathway enrichment analysis revealed that 7 pathways were enriched as follows: pathways in cancer, cAMP signaling pathway, metabolic pathways, PPAR signaling pathway, TNF signaling pathway, PI3K-Akt signaling pathway, and AGE-RAGE signaling pathway in diabetic complications.

Long-term UC patients have a great risk of developing colitis-associated cancer [46], and TLR4/NF-*κ*B-, TNF-*α*-, and IL-6-related pathways are crucial in the progression of UC to colon cancer [47]. One of the most universal comorbidities of UC is diabetes mellitus. The interaction of AGE-RAGE has been demonstrated to affect the morphology and function of the gut in diabetic patients. The RAGE signaling pathway is relevant to intestinal inflammation and permeability in both CD and UC [48]. Evidence has suggested that the gut microbiota is also likely to affect UC by altering metabolism via the generation of specific enzymes and/or metabolites [49]. Because of this, UC has a strong connection to metabolic diseases and pathways. Accumulating evidence supports cAMP's role in triggering key features of inflammatory resolution, including induction of proresolving mediators, apoptosis, efferocytosis and phagocytosis, nonphlogistic recruitment of macrophages, macrophage polarization, and tissue homeostasis [50]. Peroxisome proliferator-activated receptors (PPARs) are ligand-inducible nuclear receptor transcription factors that can regulate UC. Oleoylethanolamide restores the mRNA transcription of PPAR-*α* blocked by dextran sodium sulfate (DSS) in mice colitis [51]. Activating the PPAR-*γ* pathway in the intestinal epithelium by 5-aminosalicylic acid ameliorates colitis in mice treated by DSS [52]. TNF is a key proinflammatory cytokine, and the myricetin derivative M10 inhibits necroptosis in inflamed colonic mucosal cells in mice by downregulating the TNF-*α* pathway [53]. PI3K/Akt signaling pathways have been shown to regulate and induce the release of proinflammatory cytokines, such as TNF-*α* [54]. Additionally, studies have documented that the PI3K/Akt/mTOR signaling pathway exerts a vital regulatory effect on inflammation and apoptosis of UC cells [55]. Collectively, based on the findings of this study, multiple targets and pathways, such as inflammation, immunity, cell proliferation, and apoptosis, as well as cancer, participate in the pathogenesis and progression of UC.

Subsequent analysis of the PPI results revealed that CD44, HIF1A, and MMP2 were the top 3 DEmRNAs based on the circRNA-associated ceRNA network. CD44 and HIF1A are ferroptosis-related genes in UC [56]. CD44 is a glycoprotein present on the surface of cells that participates in cell-cell interactions, adhesions, and migration [57]. CD44 not only is involved in the pathogenesis of UC [56] but also can serve as a predictor of tumor development and a therapeutic target [58]. Hypoxia leads to the activation of the hypoxia-inducible factor (HIF)-signaling pathway during inflammatory diseases [59]. Research has proven that in severe UC, cyclosporine A modulates neutrophil function directly through the SIRT6-HIF-1*α*-glycolysis axis, ameliorating intestinal mucosal damage and alleviating clinical symptoms [60]. The novel H3NT protease MMP-2 is required for myogenic gene activation and myoblast differentiation [61], and the data indicated that MMP-2 and MMP-9 levels in UC patients' serum are markedly elevated, which further exacerbates the occurrence and progression of UC [62]. Likewise, PPI network analysis showed that mRNAs based on the ceRNA network are closely connected to UC, especially in inflammation, cell proliferation, and apoptosis as well as cancer. GO, KEGG enrichment, and PPI network analyses were performed to confirm our previous hypothesis.

By exploring the circRNA-associated ceRNA network, this study provides effective evidence and a basis for refining our understanding of UC from a molecular perspective. However, this research also has the following obvious limitations: ① the limited sample size may result in the biased selection of circRNAs, miRNAs, and mRNAs, and analyses of biological processes are lacking; ② our results refer only to colonic tissue specimens, and other specimens (such as blood and tissue fluid) were not included in the study; and ③ the functions of the circRNAs and miRNAs remain poorly understood, requiring functional experiments in vivo and in vitro. In future research, we need to expand the sample size to confirm the above findings and further study other specimens to better understand UC's physiological and pathological processes. Additionally, it is necessary to conduct the corresponding circRNA interaction analysis, carry out stricter scientific experiments, and ultimately find a specific pathway in this circRNA-associated ceRNA network.

## 5. Conclusions

On the basis of the ceRNA hypothesis, we innovatively constructed a circRNA-miRNA-mRNA network, systematically analyzed and observed the network-mediated roles of genes in the occurrence and development of UC. circRNAs and mRNAs are both regulated by and negatively coexpressed with specific miRNAs; thus, the functions of circRNAs correspond to the linked mRNAs. We identified 3 miRNAs (hsa-miR-342-3p, hsa-miR-199a-5p, and hsa-miR-142-3p) as core miRNAs in the circRNA-associated ceRNA network. Additionally, the functions and potential pathways of mRNAs in the UC-related circRNA-associated ceRNA network were further illustrated using GO and KEGG enrichment analyses, as well as PPI network analysis. We further ascertained 3 hub genes (CD44, HIF1A, and MMP2) in the PPI network. Based on the results of this study, it appears that the circRNA-associated ceRNA network is engaged in the inflammatory response, immune response, cell proliferation, apoptosis, and carcinogenesis of UC.

## Figures and Tables

**Table 1:** The top 5 GO terms in each category are obtained by analyzing DEmRNAs. BP, biological process; CC, cellular component; MF, molecular function. Enrichment score: the enrichment score of GO, which is accounted by −log_10_ (*p* value).

Category	Id	Terms	Gene count	Enrichment score	*p* value
BP	GO: 0045766	Positive regulation of angiogenesis	**4**	**3.60568**	**0.09838**
BP	GO: 0071498	Cellular response to fluid shear stress	**2**	**19.35051**	**0.09784**
BP	GO: 1902287	Semaphorin-plexin signaling pathway involved in axon guidance	**2**	**19.35051**	**0.09784**
BP	GO: 0044319	Wound healing and spreading of cells	**2**	**19.35051**	**0.09784**
BP	GO: 0008285	Negative regulation of cell proliferation	**7**	**2.22298**	**0.09387**
CC	GO: 0005912	Adherens junction	**4**	**3.63691**	**0.09652**
CC	GO: 0014069	Postsynaptic density	**5**	**2.89299**	**0.09356**
CC	GO: 0030175	Filopodium	**3**	**5.80063**	**0.09347**
CC	GO: 0015629	Actin cytoskeleton	**5**	**3.01878**	**0.08309**
CC	GO: 0098982	GABAergic synapse	**3**	**6.27740**	**0.08172**
MF	GO: 0004467	Long-chain fatty acid-CoA ligase activity	**2**	**18.95455**	**0.09979**
MF	GO: 0008559	Xenobiotic-transporting ATPase activity	**2**	**18.95455**	**0.09979**
MF	GO: 0004065	Arylsulfatase activity	**2**	**20.30844**	**0.09345**
MF	GO: 0015562	Efflux transmembrane transporter activity	**2**	**20.30844**	**0.09345**
MF	GO: 0005515	Protein binding	**96**	**1.09030**	**0.09125**

**Table 2:** The total 14 KEGG pathway terms are obtained by analyzing DEmRNAs. KEGG, Kyoto Encyclopedia of Genes and Genomes.

Category	Id	Terms	Gene count	Enrichment score	*p* value
KEGG-PATHWAY	hsa05200	Pathways in cancer	**10**	**1.91667**	**0.07068**
KEGG-ATHWAY	hsa00071	Fatty acid degradation	**3**	**7.10058**	**0.06501**
KEGG-PATHWAY	hsa04024	cAMP signaling pathway	**6**	**2.76312**	**0.06304**
KEGG-PATHWAY	hsa04216	Ferroptosis	**3**	**7.44695**	**0.05976**
KEGG-PATHWAY	hsa01100	Metabolic pathways	**22**	**1.45393**	**0.05858**
KEGG-PATHWAY	hsa04610	Complement and coagulation cascades	**4**	**4.78941**	**0.04927**
KEGG-PATHWAY	hsa05418	Fluid shear stress and atherosclerosis	**5**	**3.66097**	**0.04575**
KEGG-PATHWAY	hsa05133	Pertussis	**4**	**5.35658**	**0.03728**
KEGG-PATHWAY	hsa03320	PPAR signaling pathway	**4**	**5.42800**	**0.03606**
KEGG-PATHWAY	hsa04668	TNF signaling pathway	**5**	**4.54353**	**0.02316**
KEGG-PATHWAY	hsa04151	PI3K-akt signaling pathway	**9**	**2.58750**	**0.02093**
KEGG-PATHWAY	hsa01212	Fatty acid metabolism	**4**	**7.14211**	**0.01767**
KEGG-PATHWAY	hsa04933	AGE-RAGE signaling pathway in diabetic complications	**5**	**5.08875**	**0.01595**
KEGG-PATHWAY	hsa05205	Proteoglycans in cancer	**9**	**4.46817**	**0.00079**

## Data Availability

All the data used to support the findings of this study are available from the corresponding author and can be downloaded from the NCBI-GEO database; users can download relevant data for free for the purpose of research and publish relevant articles. The datasets generated and/or analyzed during the current study are available in the GEO database (https://www.ncbi.nlm.nih.gov/geo/).

## Abbreviation

UC: Ulcerative colitis
circRNAs: Circular RNAs
miRNAs: MicroRNAs
ncRNAs: The noncoding RNA family
ceRNA: Competing endogenous RNA
NCBI-GEO: National Center for Biotechnology Information Gene Expression Omnibus
DAVID: Database for Annotation and Integrated Discovery
GO: Gene Ontology
KEGG: Kyoto Encyclopedia of Genes and Genomes
PPI: Protein-protein interaction
IBD: Inflammatory bowel disease
CRC: Colorectal cancer
DEcircRNAs: Differentially expressed circRNAs
DEmiRNAs: Differentially expressed miRNAs
DEmRNAs: Differentially expressed mRNAs
DEGs: Differentially expressed genes
BP: Biological processes
CC: Cell components
MF: Molecular functions
CD: Crohn's disease
PPARs: Peroxisome proliferator-activated receptors
DSS: Dextran sodium sulfate
mRNAs: Messenger RNAs
snoRNAs: Small nucleolar RNAs
snRNAs: Small nuclear RNAs
tRNAs: Transfer RNAs
HIF: Hypoxia-inducible factor.
